# An Interaction Network Predicted from Public Data as a Discovery Tool: Application to the Hsp90 Molecular Chaperone Machine

**DOI:** 10.1371/journal.pone.0026044

**Published:** 2011-10-11

**Authors:** Pablo C. Echeverría, Andreas Bernthaler, Pierre Dupuis, Bernd Mayer, Didier Picard

**Affiliations:** 1 Département de Biologie Cellulaire, Université de Genève, Genève, Switzerland; 2 emergentec biodevelopment GmbH, Wien, Austria; St. Georges University of London, United Kingdom

## Abstract

Understanding the functions of proteins requires information about their protein-protein interactions (PPI). The collective effort of the scientific community generates far more data on any given protein than individual experimental approaches. The latter are often too limited to reveal an interactome comprehensively. We developed a workflow for parallel mining of all major PPI databases, containing data from several model organisms, and to integrate data from the literature for a protein of interest. We applied this novel approach to build the PPI network of the human Hsp90 molecular chaperone machine (Hsp90Int) for which previous efforts have yielded limited and poorly overlapping sets of interactors. We demonstrate the power of the Hsp90Int database as a discovery tool by validating the prediction that the Hsp90 co-chaperone Aha1 is involved in nucleocytoplasmic transport. Thus, we both describe how to build a custom database and introduce a powerful new resource for the scientific community.

## Introduction

The comprehensive determination of the interactome of a protein of interest (POI) is technically challenging and in many cases impossible, even though it is ultimately indispensable to understand its functions. While there may often not be one “correct” way of screening for interactors of a POI, there is already a huge amount of data on protein-protein interactions in general from large-scale screens performed with different techniques and species. Thus, mining public databases in addition to extracting relevant information from the literature may be a more efficient approach to building a POI interactome that is reasonably reliable to serve as a discovery tool. There is clearly a need to develop a workflow to extract the data that are available for a POI but scattered across multiple databases and the scientific literature into a single virtual interactome.

Hsp90 is a highly abundant and conserved molecular chaperone that exists both in prokaryotes and in eukaryotes. The cytosolic isoforms, known for example as Hsp90α and Hsp90β in humans, are essential and have been most extensively studied [1]–[3]. Although Hsp90 has an intrinsic ATPase activity that drives its conformational changes, it really functions as a multicomponent molecular machine. A large cohort of cofactors, referred to as co-chaperones in this context, modulate many aspects of this machine including ATPase activity, recognition and selectivity, binding and release of substrates [4]. It has been speculated that the Hsp90 chaperone machine may assist up to 10% of all cytosolic proteins at some stage of their life cycle [5], but how it recognizes its substrates and, in most cases, what it does to them remain very poorly understood. Most likely because of the central role of Hsp90 in many cellular processes, cancer cells, pathogens, and viruses may be particularly dependent on it. This has led to a great interest in developing specific Hsp90 inhibitors, of which several are now in clinical trials for the treatment of cancer [6], [7].

Identifying the proteins that interact with Hsp90, either as regulators or co-chaperones or substrates (clients) is essential to understand the global functions of this essential molecular machine. A variety of biochemical and genetic efforts have been undertaken to define molecular chaperone networks more generally (for example, refs. [8], [9]–[11]) and the Hsp90 interactome specifically [12]–[22]. However, for Hsp90, the overlap between their respective hits and the number of known false negatives have been rather frustrating, most likely owing to the transient nature of many of these interactions. Further discussions of these issues and of the available approaches can be found in a very recent review [23]. Since standard proteomic or genomic approaches for this molecular chaperone machine are unable to capture the interactome comprehensively, the application of our new workflow to it appeared particularly appropriate. The specific result is a powerful discovery tool that will serve any scientific community whose paths may cross Hsp90.

## Methods

### Construction of Hsp90Int

A step-by-step protocol and scripts for building a PPI network for one's own POI(s) are provided in File S1. As indicated in the text, PPI data was retrieved and edited from public databases and the literature. For each of the seven model organisms, the data were stored in tab-delimited text files. For each pair of interacting proteins, these files contain the information about the source database, the experimental system employed to determine the interaction, and the corresponding PubMed reference(s) where available. All the PPI information contained in our text files was subjected to further processing and dynamic manipulation by conversion into visualizable PPI networks using Cytoscape 2.6.3 with a spring-embedded layout [24]. Proteins in the query list were identified and selected in each network. To detect and to extract the first level of interactors of the query list as well as interactions between these neighbors, we used Cytoscape tools “Select first neighbors of selected nodes” and “New network>from selected nodes, all edges”.

Each species-specific network was filtered in order to eliminate PPIs already described in humans by intersecting it with the human network using the Cytoscape intersection feature from the “Merge networks” tool. After converting the species-specific PPI networks into human interolog networks, we used the Cytoscape tool “Advanced network merge” to merge them into a unique network (Hsp90Int).

Note that whenever the available data did not specify which of the two cytosolic Hsp90 isoforms, Hsp90α or β, was meant, we arbitrarily assumed it was both. In general, the current datasets are too incomplete to allow a meaningful inference of isoform-specific interactomes.

### Graph measures and data evaluation

A PPI can be represented as a graph where proteins represent nodes (or vertices) and interactions represent edges. Therefore, we describe the base network of the seven model organisms as G_B_ = (V_B_,E_B_) and the network from Hsp90Int as G_H_ = (E_H_,V_H_). For G_H_ we calculated the graph measures mean degree, diameter, index of aggregation, connectivity, clustering coefficient, and assortative mixing coefficient. The graph measures were calculated with previously reported formulas [25] with partial incorporation into the JUNG graph framework (http://jung.sourceforge.net/). As a control, we generated 300 networks from G_B_ with random sets of an equal number of query vertices (nodes) from V_B_ with |V_B_| = |V_H_| and subsequent extraction of edges from G_B_ in a similar next-neighbor approach as done for the extraction of G_H_.

### Construction of functional maps

We integrated the gene ontology (GO) terms into the networks in Cytoscape by two approaches: (i) by directly associating GO annotation attributes to the members of the network using the tool import “Ontology and annotation” or (ii) by mining for over-represented GO terms in the members of the networks using the Cytoscape plugin ClueGO [26]. This plugin allows the decoding and visualization of functionally grouped GO terms in the form of networks.

### Establishment of Aha1-null fibroblasts

The generation and more detailed phenotyping of genetically Aha1-null mice will be described elsewhere. Fibroblasts from wild-type and mutant mice were established as follows. A small piece of ear was collected from an adult animal and washed in RPMI-1640 medium complemented with 30% fetal calf serum (FCS) and antibiotics. After being cut into small pieces, it was placed in 2 ml of the same medium containing 1 mg/ml collagenase and incubated overnight at 37°C. The remaining pieces were disaggregated by pipetting, and cells were collected by centrifugation. After removal of the supernatant, the cells were plated in Dulbecco's modified Eagle's medium (DMEM) with 10% FCS and antibiotics. Pools of spontaneously immortalized fibroblasts were obtained by continuous culturing.

### Antibodies

We produced a recombinant His-tagged version of mouse Aha1 in bacteria for production of a rabbit polyclonal antiserum by Stressmarq (Victoria, BC, Canada). The rabbit polyclonal serum against Hsp90α (PA3-013) was from Affinity BioReagents (Golden, CO, USA); mouse monoclonal H90-10 against Hsp90β was kindly provided by Prof. David O. Toft (Mayo Clinic, Rochester, USA); rabbit polyclonal sera against KPNA5 and IPO4 were from ProteinTech Group (Chicago, USA); the mouse monoclonal M2 against the Flag epitope was from Sigma Chemical Co. (St. Louis, MO, USA).

### Immunoprecipitations

Aha1, exportin-1 and the negative control protein GPR30 were expressed with a triple Flag tag epitope by transient transfection into HEK 293T cells. Cells were lysed for 15 min on ice in lysis buffer (20 mM Tris-HCl pH 7.4, 150 mM KCl, 0.2 mM MgCl_2_, 1 mM dithiothreitol, 2% Triton X-100). Extracts were sonicated and cleared by centrifugation at 12'000 rpm for 30 min at 4°C. 1 mg of proteins of the supernatant (in 1 ml) were incubated with anti-FLAG (M2) magnetic beads from Sigma Chemical Co. (St. Louis, MO) overnight at 4°C. After washing the immunoprecipitates with Tris-buffered saline, proteins were eluted in SDS-PAGE sample buffer without DTT by boiling. 50 mM DTT was added to the supernatants and proteins separated by SDS-PAGE and processed for immunoblotting. For reprobing the blots with a different antibody, they were stripped for 2 hours at 65°C with Tris-buffered saline containing 0.2% Tween-20.

### Nuclear localization assays

To facilitate the analysis of the nuclear localization of the glucocorticoid receptor (GR), we constructed plasmid pTom.GR for expression of a fusion protein between the red fluorescent protein Tomato [27] and the rat GR. This plasmid was transiently transfected into adult wild-type or Aha1-null fibroblasts, which were maintained in medium with charcoal-treated FCS during the experiment. Nuclear localization of this fusion protein was triggered by adding 10 nM dexamethasone. Cells were fixed in 3.7% paraformaldehyde in phosphate-buffered saline at different time points for 30 minutes. Images were taken with a Zeiss AxioCam microscope and quantitated with an image analysis software. The means of the nuclear and cytoplasmic fluorescence intensities were determined and used to calculate the nuclear/total pixel ratio.

## Results

### Building the PPI network for the human Hsp90 molecular chaperone machine

**Figure 1:**
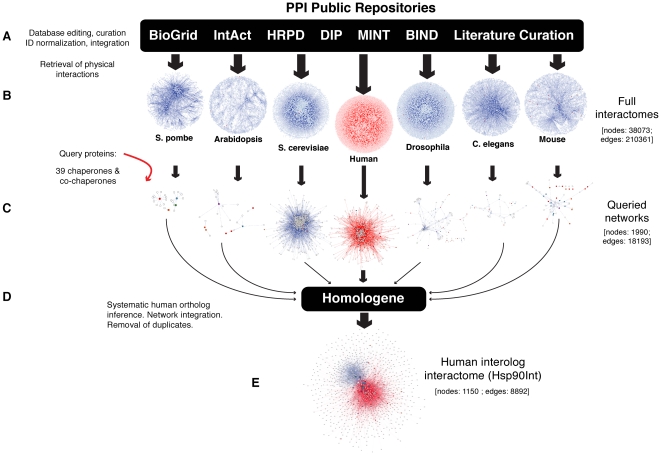
Workflow for the construction of the interactome of the human Hsp90 molecular chaperone machine (Hsp90Int). (A) PPIs from online databases were compiled and edited in order to obtain consistent and uniform data. These data were enriched with manually curated information from the literature. (B) Physical interactomes were constructed and visualized with Cytoscape for the indicated species. (C) Components of the Hsp90 molecular chaperone machine were used as query proteins (colored nodes) to retrieve the corresponding PPI network for each organism. (D) HomoloGene IDs were assigned to the interactors in each network to identify human orthologs and new interolog interactions. (E) The complete sets of interologs were merged with the human network into the complete human Hsp90Int. The red and blue colors represent human and interolog interactions, respectively.

One major difficulty in building a particular interactome from databases is that the publicly available data are deposited in several separate databases [28]. Probably owing to how the data were originally acquired, the human PPI networks coming from six different primary databases were shown to have barely any overlap. Indeed, only 3 PPIs were found in all 6 databases [29]. It is clear that the use of one or just a few databases is not enough to retrieve all known interactions of a protein, of protein complexes or entire systems. To construct the interactome of the human Hsp90 molecular chaperone machine from available data, we therefore decided to use all the major public PPI databases [29], even when the data came from different model organisms. Briefly, we built a set of species-centered PPI databases, queried them separately with a list of proteins of interest, and then merged the resulting PPI networks into a hypothetical human PPI network using the interolog concept (Figure 1). The latter proposes that, if two proteins interact in one species, identifiable human orthologs are likely to interact as well [30]. While this concept has been used in the past to build hypothetical interactomes [9], [31]–[33], to the best of our knowledge, none of these past efforts has integrated data from all major model organisms as well as from the literature, and queried the virtual interactome for a particular POI or molecular machine of interest.

We began by retrieving the full set of interactions from the main primary databases BioGrid [34], IntAct [35], HPRD [36], DIP [37], MINT [38], and BIND [39]. These included data for the budding and fission yeasts *Saccharomyces cerevisiae* and *Schizosaccharomyces pombe*, respectively, *Caenorhabditis elegans*, *Drosophila melanogaster*, *Arabidopsis thaliana*, mouse and human. To merge the data from multiple databases, they first had to be subjected to normalization and editing. This involved the following steps (Figure 1): (i) All interactions that were solely genetic as opposed to physical were discarded. (ii) We further eliminated interactions between proteins from different species. (iii) The data from each database was subdivided, where necessary, into species-centered interaction sets. (iv) Since not all databases use the official Uniprot identification number (ID) [40] or a name relating to the standard HUGO gene symbol, we had to unify the IDs with conversion tools [41]. At this point, the data could be loaded into the open source platform Cytoscape, a standalone visualization system for molecular interaction networks [24]. Thus, for each model organism, these manipulations yielded a visualizable network of all PPIs (Figure 1B). Such networks retain all of the source information including links to the original literature reports allowing one to assess the experimental evidence for a given PPI. Considering that the PPI databases only partially cover the known interactions involving the Hsp90 chaperone machine, we enriched the networks with manually curated data from the literature that we have been making available to the community for many years (see http://www.picard.ch/downloads). The mining of several hundred publications had yielded over 250 Hsp90-interacting proteins, which were not present in the public PPI databases (indicated in Table S1). The datasets were now ready for querying.

**Figure 2:**
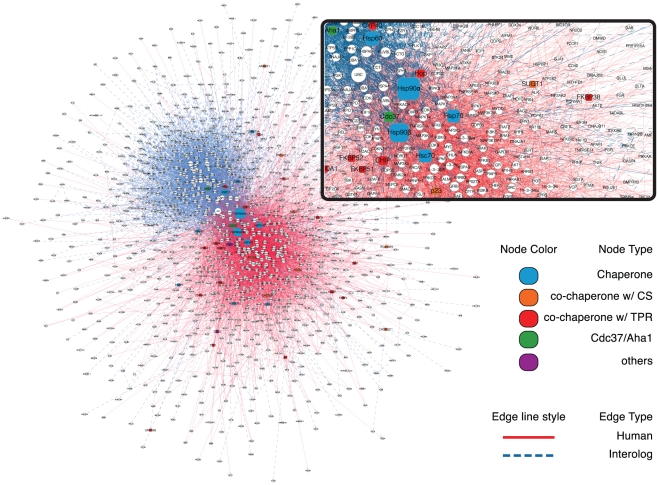
Visualization of Hsp90Int. The full interactome containing 1'150 nodes and 8'892 edges is presented here as a zoomable PPI network with query proteins but not their interactors being colored (see inset). The sizes of the nodes reflect their level of association with partners inside the network (degree). The origins of the interactions are indicated by the color of the edge line: interologs and human interactions are represented with blue dashed and red full lines, respectively. Chaperone, core molecular chaperones such as Hsp90α and Hsp90β; TPR, tetratricopeptide repeats; CS, “CHORD and Sgt1 domain”.

The Hsp90 chaperone machine does not consist of only one protein complex, but of a multitude of complexes that interconvert and that may differ in a cell type-specific and species-specific manner. In addition, some co-chaperones may have their own and even partially Hsp90-independent PPI networks. To capture this complexity, we wanted to query our database with all known components of the Hsp90 chaperone machine. This list was the result of our continuous literature mining effort mentioned above. This query list, complemented with a few additional related molecular chaperones, contained 39 proteins (Table S2) and was used to mine the seven species-centered interactomes. Both first level interactions between all query proteins and other proteins in the database and second level interactions between these primary hits were extracted to generate seven species-centered “queried networks” (Figure 1C). The networks of the non-human species were then transformed into separate human interolog networks (Figure 1D). This could be achieved using the NCBI HomoloGene database in which each protein shares the same HomoloGene ID with all its orthologs. This step also eliminated PPIs identified in non-human species where one or the other interacting protein has no human ortholog. The non-human chaperone networks were further filtered to remove duplications, and for PPIs already known in humans to discard duplicates between inferred interologs and experimentally discovered human PPIs. Finally, the seven chaperone networks could easily be merged in Cytoscape to generate the full interactome/network of the human Hsp90 molecular chaperone machine (Figure 1E), hereafter referred to as Hsp90Int (or Hsp90Int.db for the corresponding database, which will also be made publicly available at http://www.picard.ch/Hsp90Int). Thus, editing, filtering, curation, and querying led from seven species-centered interactomes with a total of 38'073 nodes and 210'361 edges (connections) to seven query networks with 1'990 nodes and 18'193 edges, and finally to a human interactome with 1'150 nodes and 8'892 edges (Figures 1 and 2). It should be emphasized that, despite all of these manipulations, Hsp90Int rests entirely on experimental data, albeit from many different sources, and that all of the relevant original information can be traced in the source files (Table S1 and File S2).

Once assembled, Hsp90Int can be explored from many angles. For example, GO terms [42] can be integrated into it to investigate functional associations (see below), or the individual interactomes for the main Hsp90 co-chaperones Cdc37, Aha1 (see below), p23, Hop, FKBP51, FKBP52 and Cyp40 can be extracted and analyzed separately. Moreover, the network can be combined with a variety of expression data. We have used the Genevestigator platform [43] to retrieve gene expression profiles along human developmental stages. After integrating them into our network, we could generate an animation to visualize the changes in abundance of interacting proteins (Video S1).

### Topological characterization of Hsp90Int

**Figure 3:**
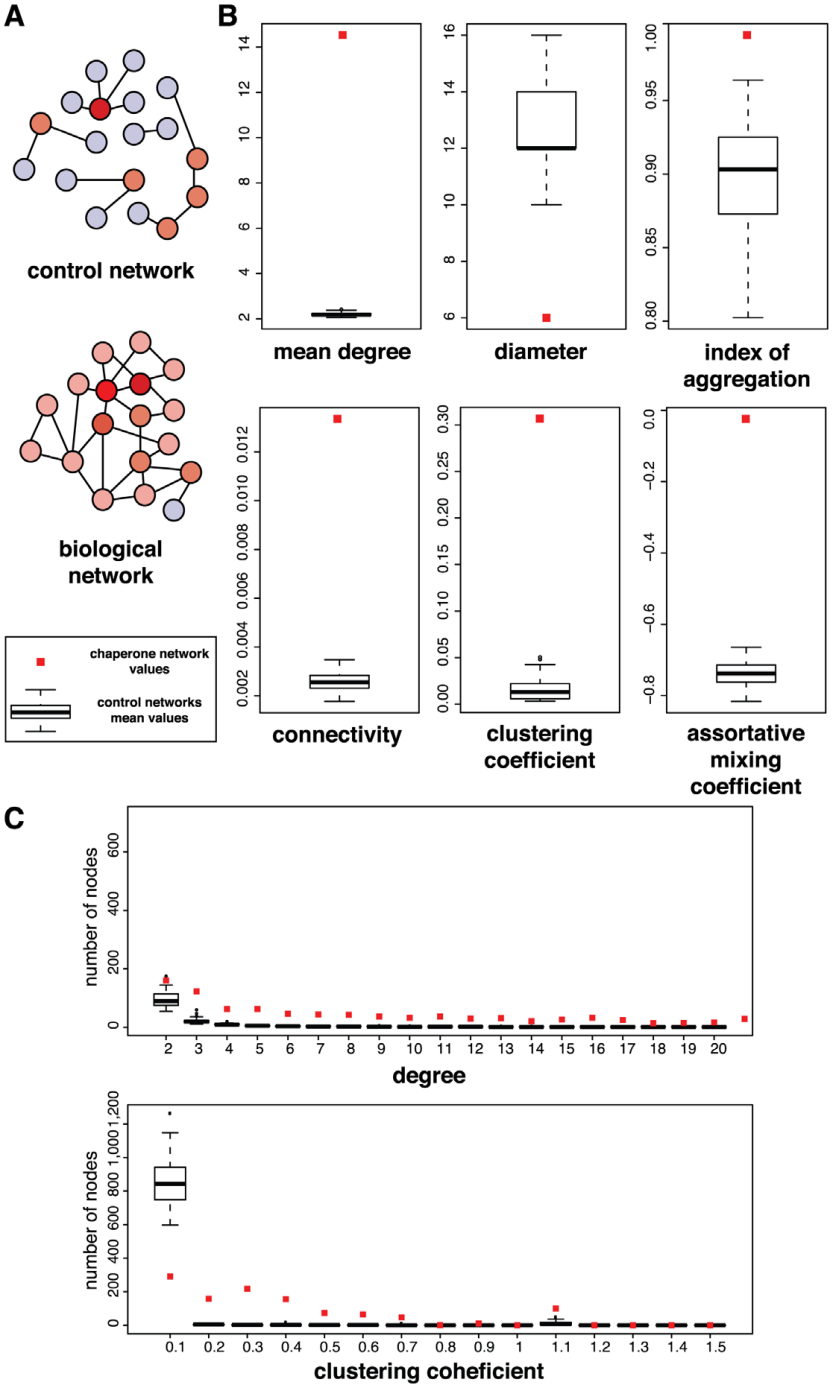
Topological characteristics of Hsp90Int. (A) Schematic representation summarizing the topological characteristics of Hsp90Int compared to randomly selected networks. (B) Mean values of the graph measures calculated for Hsp90Int (red dots) with 1'150 nodes and 8'892 edges and for the control networks (black boxplots). (C) Node distributions for mean degree and clustering coefficient.

Biological networks should be and are significantly different from random networks [44], [45]. We therefore compared Hsp90Int with 300 networks generated with different random sets of the same number of query proteins. We found that the topological properties of Hsp90Int, which are a measure of the quality and the importance of a network [46], [47], are highly significantly different from those of the control networks. The topological characteristics of Hsp90Int are depicted in Figure 3. Several graph measures were calculated and compared to a background of networks generated with randomized sets of query proteins. Significance was measured for topological characteristics such as size (diameter, index of aggregation), density (mean degree, connectivity, clustering coefficient), and distribution (assortative mixing coefficient). For example, *size* can be described by the *graph diameter*, which is the maximum distance between any two nodes, and the *index of aggregation*, which is the ratio of the size of the largest subset of proteins connected by at least one path to the total number of nodes in the network. In a *dense* network, nodes are closer to each other and connected more extensively. This is reflected by the *degree* parameter, which reports on the number of edges connected to one node.

The node degree distribution of Hsp90Int shows significantly more nodes at any degree while the distribution of the clustering coefficient shows a significantly lower number of nodes between 0 and 0.1, and significantly higher number of nodes above (especially between 0.1 and 0.7), which resembles the modular organization of metabolic networks [48]. The high density of Hsp90Int is reflected by a significantly higher mean degree, connectivity, and clustering coefficient when compared to randomly selected networks. 99% of the nodes are interconnected in the largest component, which is reflected by the high index of aggregation (0.99; control networks, μ = 0.91) and also by the lower number of connected components (6; control networks, μ = 24) whereas the diameter (6; control networks, μ = 12) highlights the compactness of Hsp90Int. The *assortative mixing coefficient* is a parameter, very well known for social networks [49], which reflects the level of preferential versus completely random associations in networks. The assortative mixing coefficient of Hsp90Int (0.02; control networks, μ = −0.74) indicates an even edge-to-edge distribution, that is a correlation between the degrees of connected nodes, which is in accordance with the dense nature of the network. Thus, the distinctive topological features of Hsp90Int distinguish it very clearly from control networks, most certainly reflecting underlying functional connections of this biological machine. This suggests that Hsp90Int.db is suitable as a discovery tool.

### Functional map of Hsp90Int

**Figure 4:**
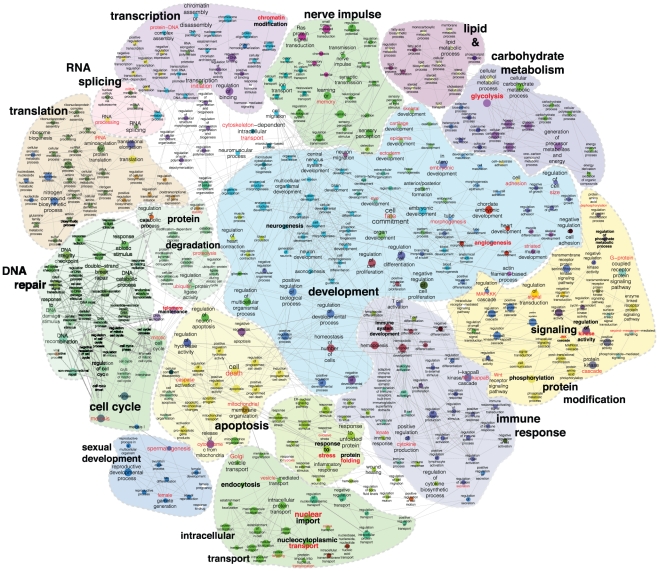
Functional map of Hsp90Int. In this functionally grouped GO annotation network, nodes represent a GO term (biological process) significantly overrepresented in a group of proteins from the interactome (at least 8). The node sizes represent the term enrichment significance. Edges connect GO terms that share common sets of proteins present in Hsp90Int. Similar functional terms are grouped in colored regions and labeled with a representative name. For clarity, the original ClueGO output was edited with Adobe Illustrator. For example, some terms are highlighted in red to guide the viewer.

One goal of determining or analyzing an interactome is to gain an overview of the biological functions of a particular protein or complex and to predict new ones. For this purpose, we investigated whether Hsp90Int contains over-represented GO terms indicating biological processes. Based on the full interactome of Figure 2, we generated a functional map (Figure 4) where members of Hsp90Int end up in nodes corresponding to their associated enriched GO terms, and where edges connecting GO terms indicate that some of their respective proteins share the same enriched GO terms. It should be noted that this functional map, like the underlying Hsp90Int, also takes advantage of and includes second level interactions between proteins that are the ones that interact with our query proteins (see Table S3 for the whole set of enriched GO terms and their associated proteins).

In the functional map, major biological functions such as development, signaling, and cell cycle cluster in distinct geographic regions that are easily identified (Figure 4). Interestingly, the functions “stress response” and “protein folding”, which could have been expected to be prominently associated with Hsp90Int, represent only a small portion of all displayed functions. “Development” is the single most extensive function on the map with development in a broad sense of many cell types, organs, and life stages being associated with the Hsp90 molecular chaperone machine itself and/or its interacting proteins that include clients and novel cofactors and regulators. Several functions on the map, for example “nucleocytoplasmic transport”, “signaling”, “cell cycle”, “immune response”, and “telomere maintenance” have previously been associated with Hsp90 and/or its co-chaperones [1], [50]–[52]. Intriguingly, the functional map offers a whole set of novel connections that may be relevant to explain the phenotype of an *hsp90α* mutation in the mouse that we have recently reported [53]. Males without Hsp90α are sterile because of a very specific pachytene arrest in spermatogenesis. The GO terms “sexual development”, “cell cycle”, and “DNA repair” are enriched and connected because of a link between the terms “spermatogenesis” and “meiosis”, respectively (Figure 4). The proteins associated with these, MLH1, MSH4, H2AFX and DMC1, would be interesting to investigate in the context of this particular phenotype.

### Functional map of the Aha1-centric PPI network

**Figure 5:**
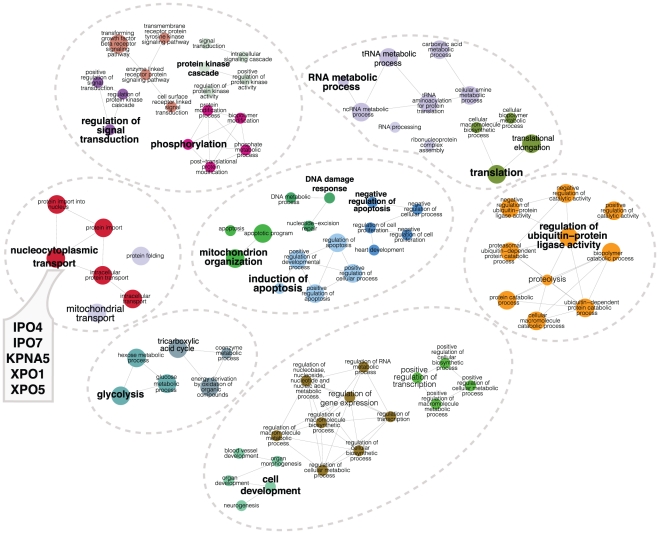
A functional map of the Aha1-Hsp90 subset. The Aha1-focused PPI network (see Figure S1) was functionally grouped into a GO annotation network. The proteins in the node “nucleocytoplasmic transport” are listed as an inset. Other map details are as in Figure 4.

To gain new insights into the functions of the Hsp90 molecular chaperone machine and at the same time to test the predictive value of our new resource, we decided to explore Hsp90Int from the point of view of the Hsp90 co-chaperone Aha1. We retrieved all of the interactors of Aha1 itself and the ones of the core chaperones Hsp90α and Hsp90β that share functional terms with Aha1 interactors (Figure S1). As before, we visualized the over-represented GO terms in a functional map for this specific interactome (Figure 5). The biological functions associated with Aha1-Hsp90 include “nucleocytoplasmic transport”. This attracted our attention because molecular chaperones have increasingly been linked to intracellular transport, either into organelles [54], [55] or into the nucleus. The latter has been highlighted recently notably for the Hsp90 complex in the context of the nucleocytoplasmic shuttling of steroid receptors [51], [56]. The proteins in the Aha1-Hsp90 functional map that share this particular GO term are listed as an inset in Figure 5. This particular GO term and the associated proteins provide the basis for our subsequent experimental validation.

### New partners and functions for Aha1

**Figure 6:**
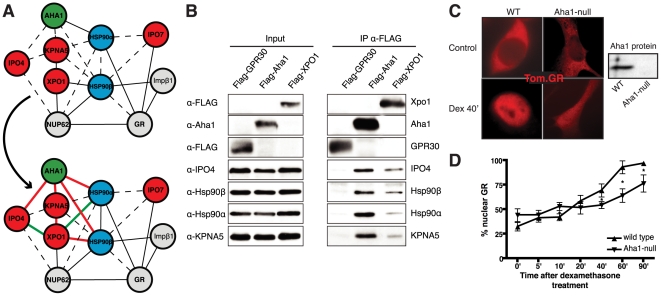
Experimental validation of the involvement of Aha1 in nucleocytoplasmic transport. (A) PPI map of the proteins (red nodes) in the GO module “nucleocytoplasmic transport” of the Aha1-Hsp90 PPI subset of Figure 5. It contains potential and known (dashed and full line edges, respectively) Aha1 interactions. The Hsp90 client protein GR was also integrated into the predicted PPI (upper panel). The co-immunoprecipitation assays of panel B allowed the experimental confirmation and new demonstration of PPIs as indicated by red and green edges, respectively (lower panel). (B) Co-immunoprecipitation experiment demonstrating interactions between Aha1 or exportin-1 (XPO1) and components of the GO module “nucleocytoplasmic transport” shown in panel A. Flag-tagged Aha1, exportin-1, and GPR30 as an unrelated control protein were exogenously expressed in 293T cells. IPO4, importin-4; KPNA5, importin-α6. (C) and (D) Nuclear localization of GR in mouse fibroblasts with and without Aha1. Panel C shows representative micrographs of the localization of Tom.GR with our without treatment with dexamethasone (Dex) for 40 min, and an immunoblot on the right verifying the absence of Aha1 in the Aha1-null fibroblasts. Panel D shows the nuclear accumulation of Tom.GR over time in wild-type (▴) and Aha1-null (▾) cells. Nuclear localization of GR was initiated at time zero with the addition of 10 nM dexamethasone. Data points are the means with standard errors of three independent experiments where ∼100 cells were counted for each time point. *, significantly different with p<0.005.

Within Hsp90Int, we zoomed in on the interactions corresponding to the module “nucleocytoplasmic shuttling” and to one particular Hsp90 client, the GR, whose nuclear localization is well known to be regulated by the Hsp90 complex [57]–[59]. Hsp90Int predicts a number of novel interactions whereas others were already more or less established (Figure 6A, upper panel). One example of the latter that also serves to illustrate some of the limitations of the data in individual public repositories is the interaction between exportin-1 (XPO1) and Hsp90, which was identified as part of an Hsp90 proteomics effort [14]. Although the Hsp90-specific antibody that was used could not have discriminated between Hsp90α and Hsp90β, only the interaction with Hsp90β found its way into some but not all databases. Overall, this close-up view from an Aha1-centric perspective predicts new PPIs and a functional role in the nuclear localization of GR. We tested the former by assessing the co-immunoprecipitation of a series of proteins with Flag-tagged Aha1 and XPO1, exogenously expressed in human 293T cells (Figure 6B). This result indicated that Aha1 and XPO1 are not only associated with both Hsp90 isoforms, but also with importin-4 (IPO4) and importin-α6 (KPNA5), thereby confirming and validating several connections (indicated as solid red edges in the lower panel of Figure 6A). Although our current data cannot discriminate between direct and indirect interactions, we speculate that most if not all Aha1 interactions discussed here are Hsp90-mediated.

To examine the functional importance of Aha1 for the “nucleocytoplasmic transport” of GR, we compared the kinetics of its hormone-induced nuclear localization in cells with and without Aha1. We transiently expressed GR as a red fluorescent protein fusion protein (Tom.GR) in mouse fibroblast cells that were either wild-type or lacking Aha1 because of a gene trap mutation (herein referred to as Aha1-null cells). Without the steroid hormone dexamethasone (Dex), Tom.GR showed a fairly similar and primarily cytoplasmic subcellular localization, both with and without Aha1. After 40 minutes of Dex treatment, Tom.GR was almost exclusively nuclear in wild-type cells but only partially nuclear in Aha1-null cells (Figure 6C). A time-course confirmed that the nuclear accumulation of Tom.GR is slower and ultimately less complete in the absence of Aha1 (Figure 6D). This quantitative analysis also revealed that the nuclear levels of the unliganded Tom.GR may be slightly higher in the absence of Aha1, possibly further supporting the conclusion that Aha1 is involved in “nucleocytoplasmic transport” and that the overall equilibrium may be perturbed in its absence.

## Discussion

We have presented a novel workflow to assemble the virtual interactome of a POI from the vast amount of data that are already available in a variety of public databases. We have applied it to the Hsp90 molecular chaperone machine where the results from individual efforts to describe the interactome have been particularly incomplete. Our approach to building Hsp90Int has proven superior to other available databases or algorithms. There are of course already a number of storehouses that combine different PPI databases by merging networks stored in different formats, even inferring human PPIs from orthologs, or working as Cytoscape plugins and allowing the mining of interactomes using a protein query list. However, for our purposes they proved to be incompletely consolidated and insufficiently updated. For example, the use of our query list to mine the human data in BisoGenet [60] yielded only 553 nodes and 3424 edges compared to 618 nodes and 4552 edges in our own human PPI data. Two features of our workflow have been critical to build Hsp90Int. We used data from several model organisms and incorporated and/or converted them into a human PPI network, and we incorporated a large amount of PPI data from the literature. As far as the workflow for building the database goes, there is nothing peculiar about the fact that this is a human PPI network except that it relies on far more primary data from the same species. This approach could be applied for any other species as well except that the proportion of predicted interactions would be much higher. An important feature is that once a PPI network has been built, it can be explored from many different angles. One can zoom into another POI or one can interrogate it with functional GO terms. And it can easily be updated, which is essential for it to remain a useful tool.

The database acronym and our presentation of Hsp90Int should not mislead to believe that it only contains interactors of Hsp90 or Hsp90 co-chaperones themselves. Even though Hsp90Int.db is strongly enriched for Hsp90 interactors because of the weight of the manually incorporated literature data, it extends beyond the core of the cytosolic Hsp90 machine by design and by necessity. Our query list also contained the Hsp90 isoforms of mitochondria and the endoplasmic reticulum, even though hardly any interactors are currently known for these proteins. It also contained Hsp70 and some of its isoforms as another major molecular chaperone. This was necessary since Hsp70 functionally collaborates and interacts, at least indirectly through Hop, with Hsp90. For newly synthesized proteins, the Hsp70 multicomponent machine tends to work upstream of Hsp90 [61], [62], and thus, in this context and others, intersections between these two molecular chaperone systems are expected.

To demonstrate the usefulness and the power of Hsp90Int.db, we followed up on the prediction that Aha1 is involved in nucleocytoplasmic transport and were able to confirm it experimentally. Naturally, these results raise further questions. For example, the interplay of Aha1 and Hsp90 will need to be further clarified. Likewise, it will be interesting to investigate the generality of a role for Aha1 in nuclear import and its role in export. However, these early successes of Hsp90Int.db should suffice to incite researchers to explore Hsp90Int.db and/or to build a PPI for their own POI using our workflow.

## Supporting Information

Figure S1 Pdf file with a visualization of the Aha1 PPI network. Interactors of Aha1 itself and the ones of the core chaperones Hsp90α and Hsp90β that share functional terms with Aha1 interactors.
(PDF)

File S1 Compressed archive containing a step-by-step protocol for building a PPI network (in pdf format) and a folder with scripts.
(ZIP)

File S2 Hsp90Int data as a Cytoscape file (xgmml format for import).
(XML)

Table S1 Excel file with the full list of interactors and interaction pairs and associated information of Hsp90Int.
(XLS)

Table S2 Pdf file with list of query proteins.
(PDF)

Table S3 Excel file listing the enriched GO terms associated with the components of Hsp90Int.
(XLS)

Video S1 Animation file in .mov format with a visualization of the dynamic changes in expression levels of components of Hsp90Int along human development.
(MOV)
