# Supplementary material for: An Interaction Network Predicted from Public Data as a Discovery Tool: Application to the Hsp90 Molecular Chaperone Machine
Source: PLoS One. 2011 Oct 11;6(10):e26044. doi: 10.1371/journal.pone.0026044 (PMC3195953; doi:10.1371/journal.pone.0026044)
Supplement: Figure S1 — Pdf file with a visualization of the Aha1 PPI network. Interactors of Aha1 itself and the ones of the core chaperones Hsp90α and Hsp90β that share functional terms with Aha1 interactors. (PDF) [file pone.0026044.s001.pdf]

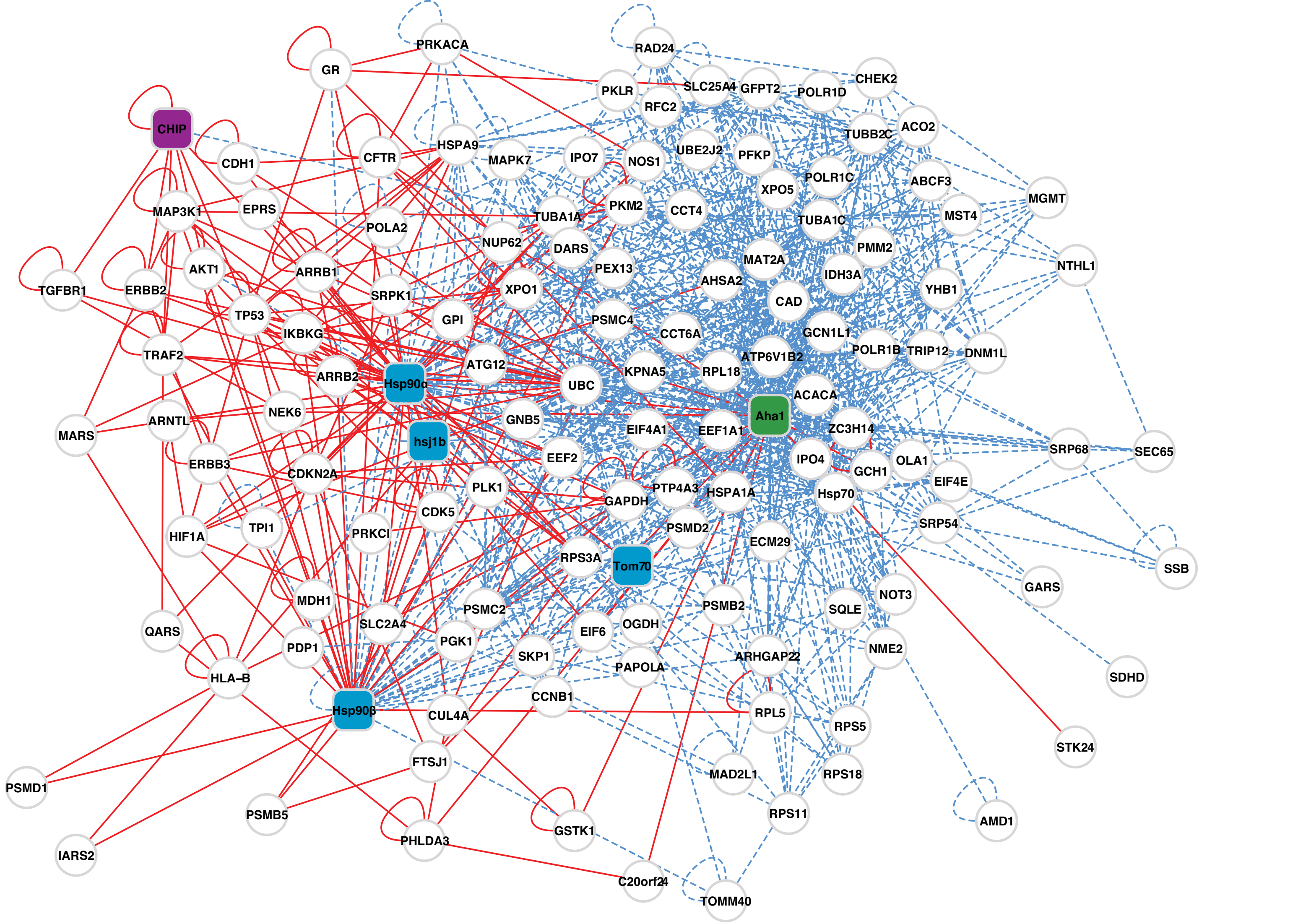

Figure S1. Aha1 PPI network. Interactors of Aha1 itself and the ones of the core chaperones Hsp90α and Hsp90β that share functional terms with Aha1 interactors. The colors, shapes and line formats are the same as in Figure 2.
