# Supplementary material for: An Interaction Network Predicted from Public Data as a Discovery Tool: Application to the Hsp90 Molecular Chaperone Machine
Source: PLoS One. 2011 Oct 11;6(10):e26044. doi: 10.1371/journal.pone.0026044 (PMC3195953; doi:10.1371/journal.pone.0026044)
Supplement: File S1 — Compressed archive containing a step-by-step protocol for building a PPI network (in pdf format) and a folder with scripts. (ZIP) [file pone.0026044.s002.zip › Protocol.pdf]

Step-by-step protocol for building your own PPI network

Obtain PPI network data

(1) Identify a list of query proteins of interest (POIs). These will be proteins, in principle, expected to function together in a cellular process and related with your specific field of research and your biological question. Be aware of the fact that these proteins may have different IDs, and stick to the names relating to the standard HUGO symbols for the genes (Eyre et al., 2006). Proteins referred to by their Uniprot accession number (Jain et al., 2009) should also be converted to the HUGO nomenclature.

(2) The folder “PPI_network” contains all the scripts that are necessary. Also make sure you have Cytoscape and Excel (or another spreadsheet application).

(3) Go to the “PPI_network” folder and there to the folder “extract_interactome”. Modify manually the “query_file” file with your own list of POIs. Make sure that you conserve the same format.

(4) Acquiring the data from multiple PPI repositories including BioGrid, BIND, HPRD, IntAct, DIP, and MINT (URL details in the following table). Before these databases can be queried, the data must be locally available. Hence, download the PPI files available for several model organisms such as budding and fission yeasts, *Caenorhabditis elegans*, *Drosophila melanogaster*, *Arabidopsis thaliana*, mouse and human. The downloaded data files can be used to make or to update the PPI data from these repositories in the folder “databases” (in the folder “extract_interactome”). The ones currently in the folder should be replaced by the newly downloaded ones, respecting the names exactly as shown to ensure that the query script will work properly (see step #5). Note that some data files will be relatively large. The script will select the physical interactions and discard other types of interactions reported in these repositories (for example, genetic interactions).

Database name	URL
**BioGrid**	http://thebiogrid.org/
**BIND**	http://bond.unleashedinformatics.com/
**HPRD**	http://www.hprd.org/
**IntAct**	http://www.ebi.ac.uk/intact/
**DIP**	http://dip.doe-mbi.ucla.edu/dip/
**MINT**	http://mint.bio.uniroma2.it/mint/

(5) Querying the databases by using the provided script. Once the “database” folder has been updated, open a Terminal window (in Mac OS or Ubuntu). Use the “cd” command to go to the folder “extract_interactome” in the “PPI_network” folder, which contains the script. Type “perl script.pl” and press enter.

(6) This script generates several files in the folder "result_files". Specifically, it creates one file for each organism in each database. If you open these text files in Excel, they might look like this:

Example file 1

Interactor_A	Interactor_B	Experimental_system	Source_database	PubMed_ID	Predicted_from
Protein1	Protein2	coIP	BioGrid_human	#########	human
Protein1	Protein3	coIP	IntAct_human	#########	human
Protein4	Protein5	surface plasmon resonance	Literature-mining_human	#########	human

Example file 2

Interactor_A	Interactor_B	Experimental_system	Source_database	PubMed_ID	Predicted_from
ProteinY	ProteinX	coIP	BioGrid-cerevisiae	#########	S.cerevisiae
ProteinY	ProteinZ	coIP	IntAct_ cerevisiae	#########	S.cerevisiae
ProteinQ	ProteinR	Yeast two hybrid	MINT_cerevisiae	#########	S.cerevisiae

(7) Mine your personal collection of papers related to your list of POIs and/or Pubmed to identify interactions that are missing from the afore-mentioned public repositories. With these data build Excel files according to the format shown above.

(8) Remember that the standard HUGO nomenclature should be used for the protein names.

Explore, manipulate and generate visible PPI networks in Cytoscape

Full details for how to work with the software Cytoscape have been previously described (Cline et al., 2007; http://www.cytoscape.org).

(9) Import your network data, from the Excel file assembled in step 4 (for your organism of interest), into Cytoscape: Under the File menu, select Import → "Network from Table (Text/MS Excel)". Locate your file and click OK.

(10) Generate a layout for your network: Use the spring-embedded layout algorithm provided by Cytoscape to explore and to visualize more easily the PPI data. This layout facilitates the identification of groups of tightly connected proteins, potentially associated with functional modules, and "hub proteins", which interact with many other proteins and often represent functionally crucial/central proteins. Export the constructed networks as a file for future use. Under the File menu, select Export → "Network and attributes as XGMML".

(11) Identify and focus on any particular protein in the network: Go to the “Search” field at the center of the Cytoscape toolbar and type the name of the protein and then click the enter key. This action will select the protein in the network. To

select its interaction partners as well go to the Select menu and select Nodes → "First neighbors of selected nodes". All selected proteins can be extracted into a new network (File menu, select New → Network → "From selected nodes, all edges") for a better visualization or analysis of the PPI network of the selected protein.

Predict new interactions and functions from the network data

(12) Identify interolog interactions: Interologs are orthologous pairs of interacting proteins in different organisms. Once the PPI network of your list of POIs in one organism of interest has been defined, it is possible to assume that the orthologous interactions in other organisms could serve to predict further interactions.

(i) To merge multiple interolog networks into one for your organism of interest, make use of the Homologene database (http://www.ncbi.nlm.nih.gov/sites/entrez?db=homologene). In Homologene, each group of orthologous proteins has the same ID (HID). Download these data from the Homologene ftp site (ftp://ftp.ncbi.nih.gov/pub/HomoloGene/). Using this dataset you will be able to build Excel spreadsheets where the list of the standard HUGO gene symbols will be associated to a unique HID and to one organism (NCBI Taxonomy ID) in a case-sensitive match. Your Excel file might look like this:

Gene Symbol	HID	Taxonomy ID
APOH	26	9606
APOH	26	9615
APOH	26	9913
Apoh	26	10090
Apoh	26	10116
fas	27	7955
FAS	27	9031
FAS	27	9598
FAS	27	9606

(ii) Split your Excel file into different Excel files for each organism under analysis maintaining the association between Gene Symbol and HID (e.g. the Taxonomy ID 9606 corresponds to the organism Homo sapiens).

(iii) Integrate the HID data into the networks generated in step 5: Go to the File menu → Import → "Attribute from Table (Text/MS Excel)". Verify that the HID attribute has been loaded by returning to the Node Attribute Browser and clicking the Select Attribute button.

(iv) Load all the different organism networks that you want to combine in Cytoscape, one by one, using File → Import → "Network (multiple file types)".

(v) Merge all these networks: in the menu Plugins → "Advanced Network Merge", once the dialog box is open, add all the networks to the right panel

(using the arrow buttons). Select a matching attribute for each network, in this case HID, which is the one all the orthologous proteins will have in common. If you select the option "Union", matched nodes will be merged into one in the resulting network.

(vi) Select the predicted interactions from the "Edge Attribute Browser" tab at the bottom of the Data Panel and the attribute "predicted from" to display. The predicted interactions will be the ones derived from the other organisms, i.e. the interologs (e.g. interactions known in *Saccharomyces cerevisiae*, *Arabidopsis*, and mouse, and predicted for human if that's the organism you focus on).

(vii) After selecting the "predicted from" edges with the Select menu, select Nodes → "Nodes connected by selected edges". All the new predicted interactions and proteins will be selected and ready to analyze.

Detect enriched protein functions

(13) Load the Gene Ontology (GO) annotations into your networks in Cytoscape: Go to File → Import → "Ontology and Annotation". Select "Gene Association file" for a corresponding organism. Retrieve the GO data.

(14) Using the Cytoscape "Enhanced Search Plugin" (ESP), you will be able to select all the proteins with particular key words in their GO terms. Simply type the key words in the ESP search field (e.g. "chromatin modification").

(15) Use the Cytoscape plugin ClueGO to assess if the proteins in your network are enriched for any biological processes recorded in the GO database (Ashburner et al., 2000). Further details are available in the original ClueGO publication (Bindea et al., 2009).

References

Ashburner, M., Ball, C.A., Blake, J.A., Botstein, D., Butler, H., Cherry, J.M., Davis, A.P., Dolinski, K., Dwight, S.S., Eppig, J.T. et al. (2000). Gene ontology: tool for the unification of biology. The Gene Ontology Consortium. Nat. Genet. *25*, 25-29.

Bindea, G., Mlecnik, B., Hackl, H., Charoentong, P., Tosolini, M., Kirilovsky, A., Fridman, W.H., Pages, F., Trajanoski, Z., and Galon, J. (2009). ClueGO: a Cytoscape plug-in to decipher functionally grouped gene ontology and pathway annotation networks. Bioinformatics *25*, 1091-1093.

Cline, M.S., Smoot, M., Cerami, E., Kuchinsky, A., Landys, N., Workman, C., Christmas, R., Avila-Campilo, I., Creech, M., Gross, B. et al. (2007). Integration of biological networks and gene expression data using Cytoscape. Nat. Protoc. *2*, 2366-2382.

Eyre, T.A., Ducluzeau, F., Sneddon, T.P., Povey, S., Bruford, E.A., and Lush, M.J. (2006). The HUGO gene nomenclature database. Nucleic Acids Res. *34*, D319-321.

Jain, E., Bairoch, A., Duvaud, S., Phan, I., Redaschi, N., Suzek, B.E., Martin, M.J., McGarvey, P., and Gasteiger, E. (2009). Infrastructure for the life sciences: design and implementation of the UniProt website. BMC Bioinformatics *10*, 136.
