# Supplementary material for: An Interaction Network Predicted from Public Data as a Discovery Tool: Application to the Hsp90 Molecular Chaperone Machine
Source: PLoS One. 2011 Oct 11;6(10):e26044. doi: 10.1371/journal.pone.0026044 (PMC3195953; doi:10.1371/journal.pone.0026044)
Supplement: Table S2 — Pdf file with list of query proteins. (PDF) [file pone.0026044.s005.pdf]

Table S2. Query proteins. TPR, co-chaperone containing tetratricopeptide repeats; CS, co-chaperone containing a "CHORD and Sgt1" domain.

Official gene name	Common name	Member type	Uniprot ID	Extended Name	PubMed ID
HSP90AA1	Hsp90α	chaperone	P07900	Heat shock protein HSP 90-alpha	12475174
HSP90AB1	Hsp90β	chaperone	P08238	Heat shock protein HSP 90-beta	12475174
TRAP1	TRAP1	chaperone	Q12931	Heat shock protein 75 kDa, mitochondrial	16269234
HSP90B1	Grp94	chaperone	P14625	Endoplasmin	16269234
HSPA1A/B	Hsp70	chaperone	P08107	Heat shock 70 kDa protein 1A/1B	12475174
HSPA8	Hsc70	chaperone	P11142	Heat shock cognate 71 kDa protein	9269769
HSPA2	Hsp72	chaperone	P54652	Heat shock-related 70 kDa protein 2	18772114
HSPD1	Hsp60	chaperone	P10809	60 kDa heat shock protein, mitochondrial	18984579
DNAJB1	Hsp40	chaperone	P25685	DnaJ homolog subfamily B member 1	12475174
GRPEL1	hsj1b	chaperone	Q9HAV7	GrpE protein homolog 1, mitochondrial	10983842
TOMM70A	Tom70	chaperone	O94826	Mitochondrial import receptor subunit TOM70	12526792
CDC37	Cdc37	cochaperone	Q16543	Hsp90 co-chaperone Cdc37	12475174
AHSA1	Aha1	cochaperone	O95433	Activator of 90 kDa heat shock protein ATPase homolog 1	12504007
FKBP5	FKBP51	TPR	Q13451	Peptidyl-prolyl cis-trans isomerase FKBP5	12475174
FKBP4	FKBP52	TPR	Q02790	Peptidyl-prolyl cis-trans isomerase FKBP4	12475174
PPP5C	PP5	TPR	P53041	Serine/threonine-protein phosphatase 5	10400612
STUB1	CHIP	TPR	Q9UNE7	STIP1 homology and U box-containing protein 1	12475174
FKBPL	Wisp39	TPR	Q9UIM3	FK506-binding protein-like	1734870
UNC45B	UNC45	TPR	Q8IWX7	Protein unc-45 homolog B	11809970
STIP1	Hop	TPR	P31948	Stress-induced-phosphoprotein 1	12475174
S100A1	S100A1	TPR	P23297	Protein S100-A1	14638689
AIP	Xap2	TPR	O00170	AH receptor-interacting protein	19375531
FKBP8	FKBP38	TPR	Q14318	Peptidyl-prolyl cis-trans isomerase FKBP8	17024179
TTC4	Ttc4	TPR	O95801	Tetratricopeptide repeat protein 4	9819421
PPID	CYP40	TPR	Q08752	Peptidyl-prolyl cis-trans isomerase D	12475174
SVIP	Vcp/p97	TPR	Q8NHG7	Small VCP/p97-interacting protein	17785525
PIH1D1	NOP17	TPR	Q9NWS0	PIH1 domain-containing protein 1	17348703
TTC1	TPR1	TPR	Q99614	Tetratricopeptide repeat protein 1	17348703
DNAJC7	TPR2	TPR	Q99615	DnaJ homolog subfamily C member 7	17348703
NASP	Nasp	TPR	P49321	Nuclear autoantigenic sperm protein	15533935
PTGES3	p23	CS	Q15185	Prostaglandin E synthase 3	12475174
PTPLAD1	BIND1	CS	Q9P035	Protein tyrosine phosphatase-like protein PTPLAD1	18160438
CHORDC1	CHORDC1	CS	Q9UHD1	Cysteine and histidine-rich domain-containing protein 1	15642353
SUGT1	SUGT1	CS	Q9Y2Z0	Suppressor of G2 allele of SKP1 homolog	17348703
ITGB1BP2	Melusin	CS	Q9UKP3	Integrin beta-1-binding protein 2	18474241
AARSD1	AARSD1	CS	Q9BTE6	Alanyl-tRNA editing protein Aarsd1	unpublished
CACYBP	SIP	CS	Q9HB71	Calcyclin-binding protein	15271357
NUDC	NUDC	CS	Q9Y266	Nuclear migration protein nudC	17348703
CUL5	Cullin5	other	Q93034	Cullin-5	19933325
